# Effectiveness of *Opuntia ficus indica* L. *inermis* Seed Oil in the Protection and the Healing of Experimentally Induced Gastric Mucosa Ulcer

**DOI:** 10.1155/2019/1568720

**Published:** 2019-11-15

**Authors:** Ikram Khémiri, Lotfi Bitri

**Affiliations:** Unité de Physiologie des Systèmes de Régulations et des Adaptations, Faculté des Sciences de Tunis, Université de Tunis El Manar, Campus Universitaire, 2092 Tunis, Tunisia

## Abstract

Gastric ulcer is a painful lesion of the gastric mucosa which can be disabling, or even more very serious in the case of a perforation of the stomach and internal hemorrhage. Traditional pharmacopeias have shown the efficacy of various plant extracts in the treatment of this pathology. Some extracts from *Opuntia ficus indica* (OFI) have been proven to have medicinal therapeutic benefits. The aim of this study was to investigate the preventive and curative effects of OFI seed oil extracted by cold pressing on an ethanol-induced gastric ulcer model in rats. Gastroprotective activities of the oil were assessed as pretreatments prior to ethanol gavage of Wistar rats compared to reference drugs. Two oil dose effects were tested. Ulcer and gastric parameters were measured (ulcerated areas (mm^2^), % of ulcer inhibition, gastric juice volume and pH, and mucus weight). Macroscopical and microscopical assessments of the stomachs as well as gastric biopsy histological studies were carried out. OFI oil exhibited a high efficiency in the protection of the cytoarchitecture and function of the gastric mucosa against the severe damages provoked by ethanol intake. Ulcerated areas were very significantly reduced and the % of ulcer inhibition was the highest under OFI oil pretreatment. Mucus production was stimulated, gastric juice volume was reduced, and its pH was increased. Histopathological examination of H&E-stained biopsies collected from gastric mucosae from the different experimental groups confirmed the gastroprotective efficacy of OFI oil against ethanol-induced symptoms such as inflammation and damages like bleeding, erosions, lesions, necrosis, and ulcers. Furthermore, OFI oil treatment speeded-up the reduction of the surface of ethanol-induced ulcerated areas in a dose-dependent manner, leading to a time gain in the healing process. The healing rate reached 91% on day 2 and 99% on day 3, and a complete heal was attained at the fourth day under OFI oil treatment, while ulcer areas were still partially unhealed in all the other groups. The therapeutic effects of OFI oil against gastric ulcer could be mediated by its varied bioactive compounds that we have demonstrated in the analytical study. They could act synergistically or in a delayed manner to optimize the healing process through protective antioxidant properties, as well as an antagonism against histamine H_2_-receptors, a stimulation of the signaling pathways necessary for mucus and bicarbonate production, and reduction of inflammatory processes in the gastric mucosa. Additionally, OFI oil fatty acids (especially unsaturated) and triacylglycerols contribute to the reconstruction and the repair of the cell membrane lipid bilayer during the gastric ulcer healing process.

## 1. Introduction

Gastric ulcer is considered one of the most common human gastroduodenal disorders in the world. It is a disease characterized by a painful lesional excoriation, extending at least to the muscular mucosa of the gastric and/or duodenal wall, and more rarely of the lower portion of the esophagus close to the *ostium cardiacum*. It can be disabling due to epigastralgias, even serious if the stomach is perforated, leading to internal bleeding and so to death if not urgently treated [1, 2]. The main etiologies of peptic ulcer are the imbalance between the secretion of gastric hydrochloric acid and pepsin, and/or the reduction of the cytoprotective capacities of the gastroduodenal mucosal barrier by the secretion of mucus, bicarbonate, prostaglandins, and cellular antioxidants [3, 4]. In recent years, it was well admitted that the occurrence of a peptic ulcer is mainly due to infection with *Helicobacter pylori* (*HP*) [5, 6]. On a global scale, more than half of the world's population is infested by this bacteria and this rate reaches about 80% in developing countries [7–9]. The correlation between such infection and digestive diseases such as gastric and/or duodenal ulcers, chronic erosive gastritis, stomach and/or duodenal cancer, and malt lymphomas has been reported [10, 11]. Indeed, infection of the gastric mucosa by *HP* evolves in several cases into active chronic gastritis which causes in about 50% of the cases an atrophy of the gastric wall, an intestinal metaplasia which is complicated in about 20% of the cases in dysplasia and then in gastric cancer [12]. Other factors are well known to be involved in gastric ulcer induction such as medication by nonsteroidal anti-inflammatory drugs (NSAIDs) such as acetylsalicylic acid (ASA), essentially chronic emotional stress, overconsumption of alcohol and related drinks, and tobacco smoking [13–15]. For instance, the risk of developing upper and lower gastroduodenal bleeding is high in patients treated with ASA, in the prevention or in the treatment of ischemic cardiovascular diseases [16, 17]. Although the treatment of *HP* infections has improved significantly in recent years due to the development of the antibiotic pharmaceutical industry, the prevalence of peptic ulcer morbidity has not decreased accordingly and mortality from such a pathology (2.5% to 5.8%) still remains a public health concern [18]. Conventional treatments for peptic ulcers are based on one hand on the reduction of factors that induce gastric mucosal damage, such as proton pump inhibitors (IPPs), anticholinergics, histaminic H_2_-receptor antagonists, and antacids, and on the other hand on the improvement of the protective mucosal layer by mucus production or by synthetic mucus mimetic drugs [6, 19, 20]. Drugs are not without risks and trigger short- and long-term side effects on human health. Some of these side effects are abdominal pains, headache, vertigo, arrhythmias, metabolic disorders (hypomagnesemias, Vit. B12 deficiencies, and hematopoietic changes), nephritis, bone damage, and some forms of cancer [21, 22]. That is why several researches focused on the discovery of alternative medicines or therapies, based on the much diversified natural potential of bioactive compounds in plants that would clearly be less aggressive than conventional drugs [20, 23]. Several plants have pharmacological relevance since they are used in traditional or ethnopharmacopeias. In this sense, various studies around the world have reported the gastroprotective and/or healing effects of plant extracts (flowers, stems, leaves, roots, cladodes, and mucilages) on peptic ulcer induced in animal models [24–34].

The mucous membranes are contiguous to the skin which constitute the external protective barrier of the human body. They ensure the protection of the body's internal cavities which are in relation to the external environment (respiratory, digestive, urinary, and reproductive systems). If the skin is protected by the squamous layer composed of dead keratinocytes, the internal mucous membranes are protected by mucus since the cells covering them are all living cells and are highly permeable, much more than the skin.

It is well admitted that the aim of healing skin injuries of full-thickness excisions, incisions, burns, erosions, and ulcers is to promptly close the wounds by full reepithelialization, in order to avoid microbial infections and to restore the elasticity and functionality of the extracellular dermal matrix, as well as the reformation of the skin appendages. The healing of gastric ulcers is also intended to restore the gastric epithelium and to reconstruct the extracellular matrix in order to close the wounds and also to restore all the glandular and other structures that constitute the gastric wall.

Some of the methods used for the assessment of the gastrodefensive effects of plant extracts or synthetic drugs are the pylorus ligation model [35, 36] and the peptic ulcer models induced by HCL, HCL/ethanol [37, 38], acetic acid [39], and NSAIDs [40]. The ethanol-induced ulcer model in rats or mice still remains one of the most used method in the investigation of the therapeutic and/or preventive activities of active compounds, especially those from plant extracts. Indeed, ethanol was reported to prompt severe damages to gastric and duodenal mucosa by causing disturbances in the environmental equilibrium of gastroduodenal cavities [41–47].

Some fixed vegetable oils extracted by various processes (solvent, supercritical CO_2_, and cold pressure) have been proven to heal skin wounds [48–52]. In a previous work, we showed the effectiveness of *Opuntia ficus indica* oil (OFI oil) extracted by cold pressing on the healing of full-thickness skin injury, as well as its antimicrobial effect against bacteria, yeast, and fungi [53].

The current study was devoted to the investigation of the gastroprotective as well as healing potential of OFI oil as a therapeutic against ethanol-induced peptic ulcer in rat model.

## 2. Methods and Materials

### 2.1. Sample Collection

Fruits of prickly pear *Opuntia ficus indica* were harvested in the months of July and August 2015 at the village of Zelfen, located in the Thala delegation, in the governorate of Kasserine in central Tunisia. The GPS coordinates are as follows: 35°29′08^″^N and 8°45′09^″^E at an altitude of 985 m. The climate is semiarid (BSh/BSk according to the Köppen climate classification). Winters are generally chilly and wetter with snowfalls, while summers are moderately hot. The fruits were washed with clear water and drained. Then, they were peeled manually and the seeds were isolated mechanically, washed with potable water and dried in the open air.

### 2.2. Oil Extraction

The extra virgin oil of mature prickly pears seeds was naturally extracted by first cold pressing using a mechanical machine in order to preserve the quality of its components. The oil was filtered and stored in anti-UV hermetic bottles at room temperature.

### 2.3. Reagents and Drugs

Absolute ethanol was purchased from VWR Chemicals, Prolabo (France); sucralfate (Ulcar, 1 g; Sanofi-Aventis, France) and ranitidine (Azantac, 75 mg; GlaxoSmithKline, France) were acquired from a local pharmacy. The drug doses were prepared immediately before administration to the rats.

### 2.4. Physicochemical Screening of OFI Oil Extracted by Cold Pressing

The analyses were performed at the laboratories of the National Oil Office of Tunisia (ONH).

#### 2.4.1. Physicochemical Characteristics

The analyses were carried out according to the official methods of AOAC (American Oil Chemists' Society, International). Saponification index was determined according to the Norm ISO 3657: 2013. Peroxide value was estimated in meq O_2_/kg of oil (NF T60-2201998). Refractive index was measured at 20° with an Abbe refractometer with temperature adjustment. Density was determined at 20° by gravimetry. Acid index and iodine value (g I_2_/100g) were calculated according to NF ISO 660-1996 and AOAC official method 940.28 (2013), respectively.

#### 2.4.2. Free Fatty Acid Analysis

The identification and the quantification of the free fatty acids in the *Opuntia ficus indica* seed oil were performed using the Gas-Chromatography-Flame Ionization Detection (GC-FID) method. Briefly, fatty acids were extracted and methylated according to the method described [54] and modified [55]. Fatty Methyl Esters (FAMEs) were analyzed using a gas chromatograph (HP 4890) equipped with a flame ionization detector (FID). The separation of FAMEs was performed on a Supelco wax-coated capillary column (30 m long × 0.25 mm i.d. and 0.25 *μ*m film thickness). The injection was carried out with a split/splitless capillary injector (split ratio 1 : 10) and flushed with helium as carrier gas at a flow rate of 1 mL/min. Temperatures of the column, detector, and injector were, respectively, 200, 250, and 230°C. The fatty acid relative peaks were identified by comparing their retention time's standard mixture, analyzed under the same procedure. The total area was measured and served to calculate the percentage of each area under each peak, corresponding to each fatty acid. The area under each peak was measured and the percentage expressed in regard to the total area.

The calculated oxidizability (Cox) value of the oil was calculated applying the formula based on the percentage of unsaturated C18 fatty acids, proposed by Fatemi and Hammond [56]:

(1) $$\begin{array}{c} \text{Cox value}=\frac{\left(1\times \text{C}18:1\right)+\left(10.3\times \text{C}18:2\right)+\left(21.6\times \text{C}18:3\right)}{100}. \end{array}$$

#### 2.4.3. Triacylglycerol (TAG) Analysis

The analysis of triacylglycerol (TAG) fractions in OFI oil was carried out by the official qualitative and quantitative chromatographic method of the Equivalent Carbon Number (ECN 42) [57]. 0.5 g of the oil sample was dissolved in 10 mL acetone (sample solution). A chromatograph (Shimadzu, Kyoto, Japan) equipped with a refractometer detector was employed. A Spherisorb Column (250 × 4.6 mm, 5 *μ*m particle size) from Supelco (USA) permitted the separation of the TAGs. The elution was carried out with a 50 : 50 acetone/acetonitrile mixture; the flow rate was 1.5 mL/min, the column temperature was 30°C, and the injection volume was 20 *μ*L of the sample solution. The retention times were compared to standards.

#### 2.4.4. GC-FID Phytosterol Analysis

The OFI oil sterol identification and quantification were performed by Gas Chromatography-Flame Ionization Detection (GC-FID) [58]. Sterols separated from the rest of the OFI oil components were transformed into trimethylsilyl ethers (TMSs) and then analyzed by CPG-FID on a fused silica capillary column (30 cm long, 0.3 mm inside diameter) operated isothermally at 280°C. The injector temperature was maintained at 280°C and the FID detector at 290°C. Vector gas (pure helium) linear speed was 35 cm/s. The sterol fractions were identified based on a sterol mixture of known composition analyzed chromatographically under the same conditions as the tested OFI oil sample. Alpha-cholestanol was used as an internal standard.

#### 2.4.5. Tocopherol Analysis

The identification and quantification of the tocopherol fractions were carried out by high-pressure liquid chromatography (HPLC) [59].The HPLC system consisted of a low-pressure quaternary pump HP-1050, a Rheodyne injection valve (20 mL loop), a thermostatic furnace, and a fluorescence detector RF-535 (Shimadzu, Kyoto, Japan). Separation was performed in a 250 × 4 mm particle size 5 *μ*m LiChrospher Si-60 (Merck, Darmstadt, Germany) column. The column and detector were firstly separated and then detected with a UV detector.

#### 2.4.6. Total Chlorophyll and Carotenoid Content

**Figure 1:**
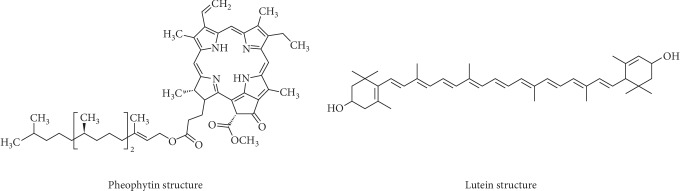
Chemical structures of pheophytin and lutein fractions. Images depict the chemical structures of major chlorophyll pigments in OI oil.

OFI oil (1.5 g) was dissolved in 5 mL cyclohexane. Chlorophyll and carotenoid amounts were determined by the colorimetric method described in [60]. The maximum absorption was measured at 670 nm which is related to the chlorophyll fraction and at 470 nm which corresponds to the carotenoid fraction.

(2) $$\begin{array}{c} \text{Chlorophyll }\left(\text{mg}/\text{kg}\right)=\frac{\left(A_{670}\times 10^{6}\right)}{\left(613\times 100\times d\right)},\\ \text{Carotenoid }\left(\text{mg}/\text{kg}\right)=\frac{\left(A_{470}\times 10^{6}\right)}{\left(2.000\times 100\times d\right)}, \end{array}$$

where “613” and “2.000” correspond, respectively, to the specific extinction coefficients of pheophytin (chlorophyll “a”, which is the major component of the chlorophyll fraction) and lutein (a xanthophyll, a major component of the carotenoid fraction) (Figure 1). “*A*” was the absorbance and “*d*” was the thickness (1 cm) of the spectrophotometer cell. The amounts of the two pigments were expressed as mg/kg OFI oil.

#### 2.4.7. Total Phenolic Content

Total phenolic content in OFI oil was quantified using the Folin-Ciocalteu method as described in [61]. An OFI oil-diluted solution (20 *μ*L) was added to 100 *μ*L of a Folin-Ciocalteu Reagent (0.2 N). The tubes were incubated at room temperature in the dark. 80 *μ*L of sodium carbonate Na_2_CO_3_ water solution (7.5%) was added to the mixture. All measurements were performed in triplicate. Absorbance was determined after 60 min at 765 nm against a blank. Total phenolics were expressed as mg of Gallic acid equivalents per g of oil (G.A. eq/g oil).

#### 2.4.8. Total Flavonoid Content

The determination of the flavonoid content in OFI oil was carried out as described in [62] but slightly modified. To an aliquot of 1.5 mL DMSO, dissolved oil was added to 1.5 mL of AlCl_3_ (2%). Absorbance was measured after 30 min incubation in the dark at 430 nm vs. a blank. Quercetin was used as standard. Total flavonoids were expressed as mg of Quercetin equivalents per g of oil (Q eq/g oil).

#### 2.4.9. Scavenging Ability against 1,1-Diphenyl-2-picrylhydrazyl Radical (DPPH Assay)

The scavenging activity towards the DPPH radical of OFI oil was evaluated using the method described in [63]. 20 *μ*L of OFI oil dilution was mixed with 180 *μ*L of 0.1 mM DPPH solution. After shaking vigorously, the mixture was left to incubate for 30 min in the dark at room temperature. The absorbance was measured at 520 nm using a spectrophotometer (Thermo Fisher Scientific Multiskan GO). The wavelength of maximum absorbance of DPPH was recorded as *A*_sample_. The absorbance of the blank control was recorded as *A*_blank_. Ascorbic acid was used as standard. All measurements were performed in triplicate.

The percentage of inhibition (%) of the free radical DPPH was calculated as follows:

(3) $$\begin{array}{c} I\%=\frac{A_{\text{blank}}-A_{\text{sample}}}{A_{\text{blank}}}\times 100. \end{array}$$

The results were expressed as Vit. C eq/g oil.

#### 2.4.10. Determination of 2,2′-Azinobis-3-ethylbenzothiazoline-6-sulfonate Free Radical (ABTS) Free Radical Scavenging Activity

An ABTS assay was carried out as described in [63]. An ABTS radical cation was produced by mixing 7 mM ABTS solution (at pH 7.4) with 2.5 mM potassium persulfate. This mixture was stored in the dark at room temperature for 16 h before use. A dilution was performed until an absorbance of 0.70 ± 0.02 at 734 nm was reached. 20 *μ*L of the sample was added to 180 *μ*L of fresh ABTS-diluted solution. Absorbance was measured 6 min after mixing. Ascorbic acid was used as standard reference. The capacity of free radical scavenging was estimated using the same equation mentioned above for the DPPH scavenging activity. The measurements were performed in triplicate. The results were expressed as Vit. C eq/g oil.

### 2.5. Experimental Study Design

#### 2.5.1. Animals

Adult male albino Wistar rats purchased from SIPHAT (Tunis, Tunisia) weighing from 180 to 190 g were acclimated for 2 weeks in the laboratory under environmentally controlled conditions: temperature of 22 ± 2°C, 12 h light/12 h dark daily artificial cycle, and 74 ± 2% air humidity. They were allowed a standard commercial pellet diet and potable water *ad libitum*. The animals were handled accordingly to the current guidelines of the Tunisian Society for the Care and the Use of Laboratory Animals (ATSAL), and the protocol for rat studies was approved locally by the Institutional Animal Ethics Committee for Animal Care and Use for scientific purposes.

#### 2.5.2. Acute Toxicity of *Opuntia ficus indica* Seed Oil (OFI Oil)

A total of 30 adult rats were divided into three groups (*n* = 10). The first group served as control. The second and the third groups were, respectively, administered *per os* (p.o.) 3.5 mL and 7 mL of OFI oil/kg/body weight (bw). They were observed for symptoms of toxicity like ataxia, convulsion, irregular respiration, diarrhea, hind legs paralysis, or mortality during 5 days.

#### 2.5.3. Gastroprotective Investigation Procedure

The antiulcerogenic effects of OFI oil were investigated using the ethanol-induced ulcer model in rats as described in [64]. The rats were fasted for 48 hours before experiments. Then, they were divided into 6 groups of ten animals each:

- Group 1: negative control group, was administered by intragastric gavage (*ig*) 1 mL of normal saline
- Group 2: positive control group, was administered by *ig* 1 mL of normal saline and one hour later 1 mL of absolute ethanol
- Group 3: sucralfate group, pretreated by *ig* with 1 g/kg/bw of sucralfate (Ulcar, 1 g) and one hour later 1 mL of absolute ethanol
- Group 4: ranitidine group, pretreated by *ig* with 50 mg of ranitidine (Azantac, 75 mg)/kg/bw and one hour later 1 mL of absolute ethanol
- Group 5: dose 1 oil group, pretreated by *ig* with 3.5 mL of OFI oil/kg/bw and one hour later 1 mL of absolute ethanol
- Group 6: dose 2 oil group, pretreated by *ig* with 7 mL of OFI oil/kg/bw and one hour later 1 mL of absolute ethanol

After one hour to ethanol exposure, the stomachs were removed from each rat, cut along the great curvature, and internally inspected. Gastric ulcerated area surface, ulcer index, percentage of inhibition of ulceration, gastric mucus weight, and volume and pH of the gastric juice were determined.

The gastric ulcerated area surface was calculated in mm^2^ by planimetry, tracing all the ulcerated areas on a transparent graph paper, then summing them. The ulcer index (UI) was estimated as described in [64]. UI was evaluated as the mean ulcerated areas (mm^2^). The percentage of inhibition was calculated using the following formula [65]:

(4) $$\begin{array}{c} \%\text{inhibition}=\left[\left(\text{UI positive control}–\text{UI tested group}\right)–\text{UI positive control}\right]\times 100. \end{array}$$

The mucus covering the gastric mucosa of each rat was gently scraped using a clean glass slide, then weighed using a sensitive balance, and the gastric volume measured using a graduated test tube as described in [27]. Gastric juice acidity was measured using a digital pH meter. A gross assessment of the state of the stomachs was carried out by photographing the upper surface of the gastric mucosae with a Sony DSC-W270 digital camera.

#### 2.5.4. Gastric Mucosa Ulcer Healing Effect of OFI Oil

The induction of peptic ulcer was undertaken using the absolute ethanol *ig* gavage described above. One hour after inducing gastric mucosa ulcer, the animals were divided into five groups of 50 each and treated as follows:

- Group 1: positive control group, not treated
- Group 2: sucralfate group, treated by *ig* once/day with 1 g/kg/bw of sucralfate (Ulcar, 1 g)
- Group 3: ranitidine group, treated *by ig* once/day with 50 mg/kg/bw of ranitidine (Azantac, 75 mg)
- Group 4: dose 1 oil group, treated *by ig* once/day with 3.5 mL of OFI oil/kg/bw
- Group 5: dose 2 oil group, treated *by ig* once/day with 7 mL of OFI oil/kg/bw

Every day 10 animals from each group were sacrificed. Their stomachs were quickly removed, opened along the great curvature, and the ulcerated areas measured as described above. Microphotographs of the scars were taken with a microscope. The remaining animals from each group were treated with their respective treatments until the next day, and so on until the end of the treatment period which was of five days till the complete healing of the mucosa ulcer of one of the groups.

#### 2.5.5. Histopathological Examination of Gastric Mucosa

Tissue specimens taken from 3 parts of the stomachs (cardia, fundus, and antrum) from the experimental animal groups were immersed in neutral-buffered formalin solution (10%) and dehydrated through a series of alcohol-water solutions using a Shandon tissue processor (Citadel 2000). After being cleared, the tissues were embedded in paraffin wax. The sections (5 *μ*m thick), made using a rotatory microtome, were mounted on a microscope glass slide and stained with hematoxylin-eosin solution (H&E). The tissue preparations were examined microscopically under light and photographed with a light Olympus microscope (Tokyo, Japan) equipped with a digital camera.

### 2.6. Statistical Analysis

Statistical data analysis was performed using SPSS statistical package (version 20.0), followed by *t*-test. The results were presented as the mean ± SEM. Significance of difference between the groups was accepted if *p* < 0.05.

## 3. Results

### 3.1. Physicochemical Screening of OFI Oil

#### 3.1.1. Physicochemical Characteristics

Table 1 presents the physicochemical characteristics of OFI oil. It is a greenish-yellow-colored, noncomedogenic, dry, and slightly fruity oil. It has a density of 0.931 ± 0.010 at 20°, an acid index of 1.952 ± 0.035, an iodine index of 108.52 ± 0.250 (g I_2_/100g oil), a peroxide index of 2.230 ± 0.061 (meq O_2_/kg oil), a saponification index of 171.40 ± 0.430 (mg KOH/g oil), and a refractive index at 20° of 1.475 ± 0.001.

#### 3.1.2. Fatty Acid Composition

**Figure 2:**
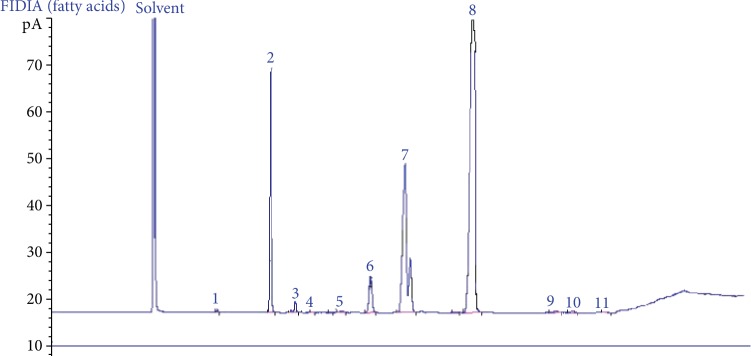
Chromatographic profile spectra of the fatty acids in the OFI oil. Data analysis of the chromatographic profile of free acids in OFI oil indicates the following composition according to their appearance on the graph: 1—C14 : 0; 2—C16 : 0; 3—C16 : 1; 4—C17 : 0; 5—C17 : 1; 6—C18 : 0; 7—C18 : 1; 8—C18 : 2; 9—C18  :  3 *n*-3; 10—C20 : 0; 11—C22 : 0.

The chromatography profile of the free fatty acid composition in OFI oil extracted by cold pressing (Figure 2) showed that the main FA were linoleic acid (C18 : 2 *n*-6), oleic acid (C18 : 1 *n*-9), palmitic acid (C16 : 0), and stearic acid (C18 : 0) with percentages (g/100 g of total fatty acids) of 61.637 ± 0.068, 21.183 ± 0.064, 12.243 ± 0.023, and 3.340 ± 0.030, respectively. The % of unsaturated fatty acids (UFA) was of 83.95 ± 0.03, that of polyunsaturated fatty acids (PUFA) was 61.87 ± 0.06, and the ratios of UFA/SFA and PUFA/UFA were 5.23 ± 0.11 and 73.69 ± 0.06, respectively. The Cox value was 6.61 ± 0.005 (Table 2).

#### 3.1.3. Triacylglycerol Composition

The different categories of TAGs in OFI oil analyzed on the basis of the equivalent carbon number method (ECN 42) are presented in Table 3. Data analysis confirmed that the major fatty acids in OFI seed oil was linoleic, oleic, and palmitic acids. The positional distribution of FA in TAG indicates PUFA linoleic acid C18 : 2 *n*-6 as the dominant homogeneous specie LLL (24.65 ± 0.029% of total TAG), followed by OLL (22.246 ± 0.015% of total TAG), PLL (17.523 ± 0.003% of total TAG), and SLL (9.94 ± 0.006% of total TAG) and OOL (9.23 ± 0.006% of total TAG). Other TAG species were identified at lower rates.

#### 3.1.4. Phytosterol Composition

**Figure 3:**
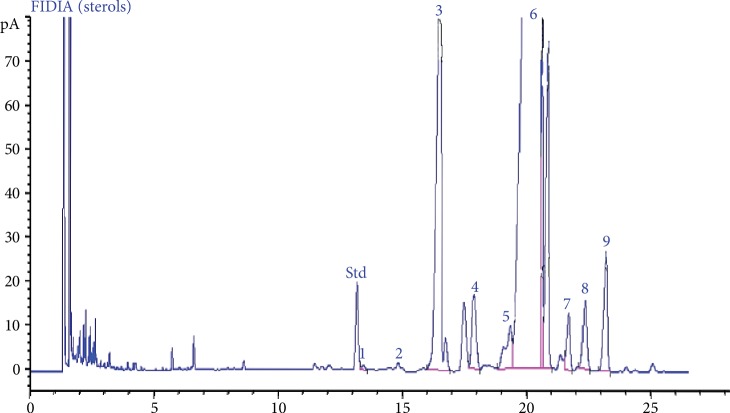
Chromatographic profile spectra of the sterols in the OFI oil obtained by GC-FID. GC-FID chromatographic profile indicates the following OFI oil composition in phytosterols according to their appearance on the graph: 1—cholesterol; 2—brassicasterol; 3—campesterol; 4—stigmasterol; 5—clerosterol; 6—*β*-sitosterol; 7—*Δ*-5,24-stigmastadienol; 8—*Δ*-7-stigmastenol; 9—*Δ*-7-avenasterol. *α*-Cholestanol was used as standard.

Figure 3 depicts the chromatographic profile spectra of the sterols in OFI oil obtained by GC-FID. Phytosterol fractions identified and evaluated in OFI oil as % of total sterols are presented in Table 4. The major sterol fraction was *β*-sitosterol (81.280 ± 0.115), followed by campesterol (11.043 ± 0.185). Other sterols were detected at low levels such as *Δ*-7-avenasterol (2.21 ± 0.005), stigmasterol (1.576 ± 0.185), clerosterol (1.417 ± 0.003), *Δ*-7-stigmastenol (1.353 ± 0.003), and *Δ*-5-24-stigmastadienol (0.963 ± 0.067).

#### 3.1.5. Vitamin E Tocopherol Composition

Vitamin E tocopherol fractions were estimated in mg/kg oil as follows: *β*- and *γ*-tocopherols (797.8 ± 0.79), *δ*-tocopherol (53.92 ± 0.46), and *α*-tocopherol (11.49 ± 0.27). These fractions from total tocopherols were, respectively, 92.422%, 6.246%, and 1.331%. The results are shown in Table 5.

#### 3.1.6. Total Phenolic, Flavonoid, Carotenoid, and Chlorophyll Contents

Our findings indicated that OFI oil has a total phenolic content of 26.5 Gallic acid eq/g oil, flavonoid content of 3.1 mg Quercetin eq/g oil, carotenoid content of 10.520 mg/kg oil, and total chlorophyll content of 4.57 mg/kg oil (Table 6).

#### 3.1.7. Scavenging Activity against DPPH and ABTS Free Radicals

Table 7 presents the data of the scavenging activity of OFI oil against DPPH and ABTS as percentage of inhibition compared to Vitamin C. We registered, respectively, 88.410 ± 0.59 and 87.420 ± 0.11 Vit. C eq/g oil.

### 3.2. Acute Toxicity of *Opuntia ficus indica* Seed Oil

In this study, we did not notice any toxicity symptoms or mortality in the orally treated animals neither with the dose of 3.5 mL nor with dose of 7 mL of OFI oil/kg/bw over the five days of the experimental period.

### 3.3. Protective Effect Study of OFI Oil against Ethanol-Induced Ulcer

**Figure 4:**
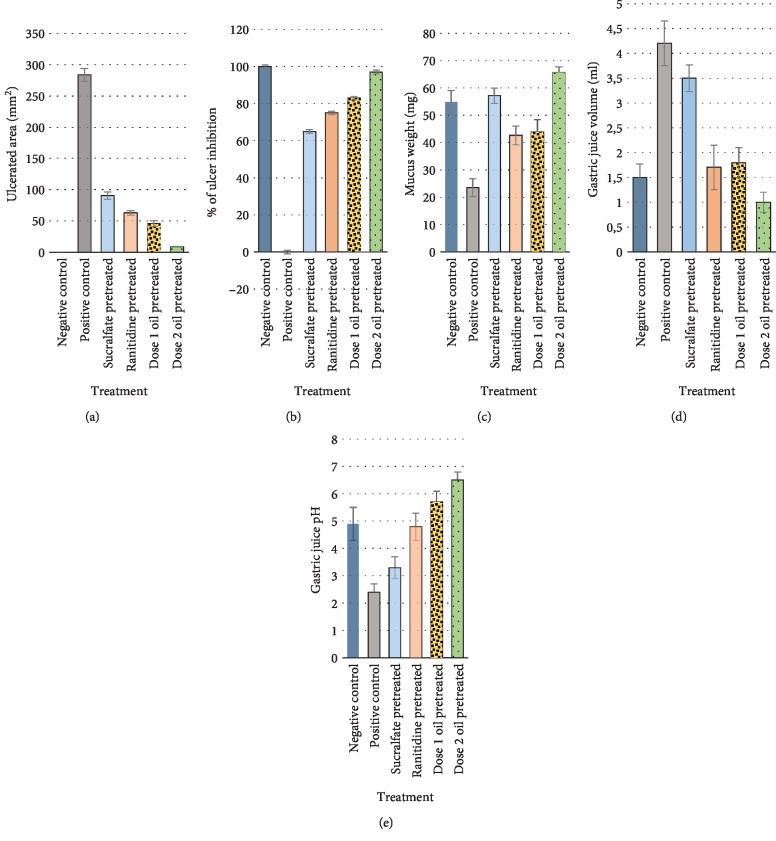
Effects of the pretreatments on ulcer (a, b) and gastric parameters (c, d, and e) in ethanol-induced gastric ulcers in rats. Graphs represent the impact of absolute ethanol on ulcer and gastric parameters and the effects of the pretreatments in preventing gastric mucosae damages.

Figure 4 shows the graphs of the effects of the pretreatments on ulcer and some gastric function parameters.

#### 3.3.1. Assessment of Ulcer Parameters

Our data indicated that absolute ethanol intake (positive control) provoked a wide range of ulceration in gastric mucosa. Sucralfate pretreatment significantly reduced ulcer areas (*p* < 0.001) vs. positive control. The same result was noted with ranitidine. The protective effect with ranitidine was significantly better than that of sucralfate (*p* < 0.01). Percentage of ulcer inhibition was significantly higher with OFI oil (dose 1) vs. sucralfate and ranitidine (*p* < 0.01). OFI oil dose 2 was the most efficient in reducing the ulcerated area surface by 97% (*p* < 0.001).

#### 3.3.2. Assessment of Gastric Parameters

Absolute ethanol intake caused a significant reduction of mucus secretion (*p* < 0.001). This was prevented by the pretreatment with sucralfate (*p* < 0.001). Ranitidine and OFI oil dose 1 effects were quite similar in enhancing mucus weight and remained lower than that of sucralfate (*p* < 0.01). OFI oil dose 2 appeared to be the most efficient in enhancing mucus secretion (*p* < 0.001).

Ethanol intragastric gavage significantly enhanced (*p* < 0.001) gastric juice volume compared to negative controls, ranitidine, and OFI oil dose 1 and 2 pretreatments, while sucralfate pretreatment slightly reduced this volume (*p* < 0.05). Gastric juice pH significantly decreased under alcohol intake vs. negative control (*p* < 0.001). This pH decrease was prevented preferentially by OFI oil dose 2, OFI oil dose 1, ranitidine, and sucralfate.

#### 3.3.3. Macroscopic and Microscopic Examination of the Gastric Mucosa after Absolute Ethanol Intake

**Figure 5:**
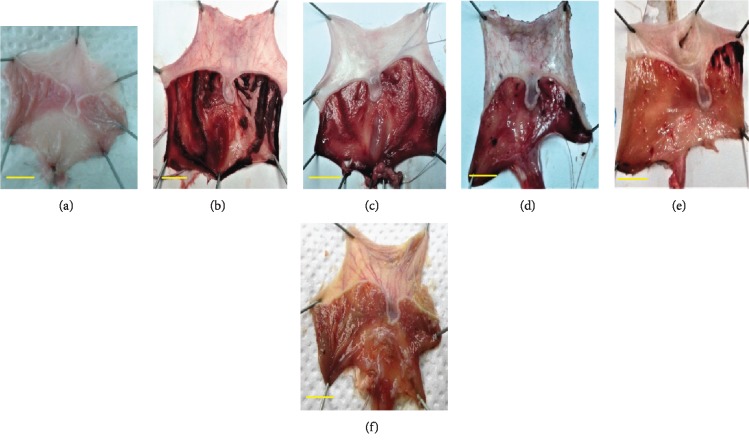
Macroscopic assessment of the pretreatment on gastric mucosa ethanol-induced ulcer: negative control (a), positive control (b), sucralfate pretreated (c), ranitidine pretreated (d), dose 1 oil pretreated (e), and dose 2 oil pretreated (f). Scale bars on the photos indicate 10 mm. Images show gross assessment of the stomachs of the different experimental groups. Ulcer degree induced by ethanol was alleviated in a crescent mode by sucralfate, ranitidine, OFI oil dose 1, and OFI oil dose 2.

*(1) Macroscopic Examination of the Gastric Mucosa*.  Figure 5 shows the photographs of the opened stomachs one hour after ethanol-induced peptic ulcer of the different experimental groups. Pretreatments with reference drugs sucralfate and ranitidine, as well as OFI oil (doses 1 and 2) were applied 1 hour prior to alcohol intake. We could observe the corrosive effects of absolute ethanol on gastric mucosa compared to negative control. Linear longitudinal and local black lesions indicate severe tissue necrosis, and redness in the remaining areas indicates vasodilation and inflammation. Sucralfate pretreatment alleviated ethanol-induced damages with linear longitudinal red lesions and hyperhaemia on the whole gastric mucosa surface. Ranitidine pretreatment reduced ethanol aggression on gastric mucosa. Local ulcers, erosions, and redness were registered. OFI oil pretreatment significantly alleviates alcohol-induced damages in a dose-dependent manner, thus ensuring a gastric mucosa protection.

**Figure 6:**
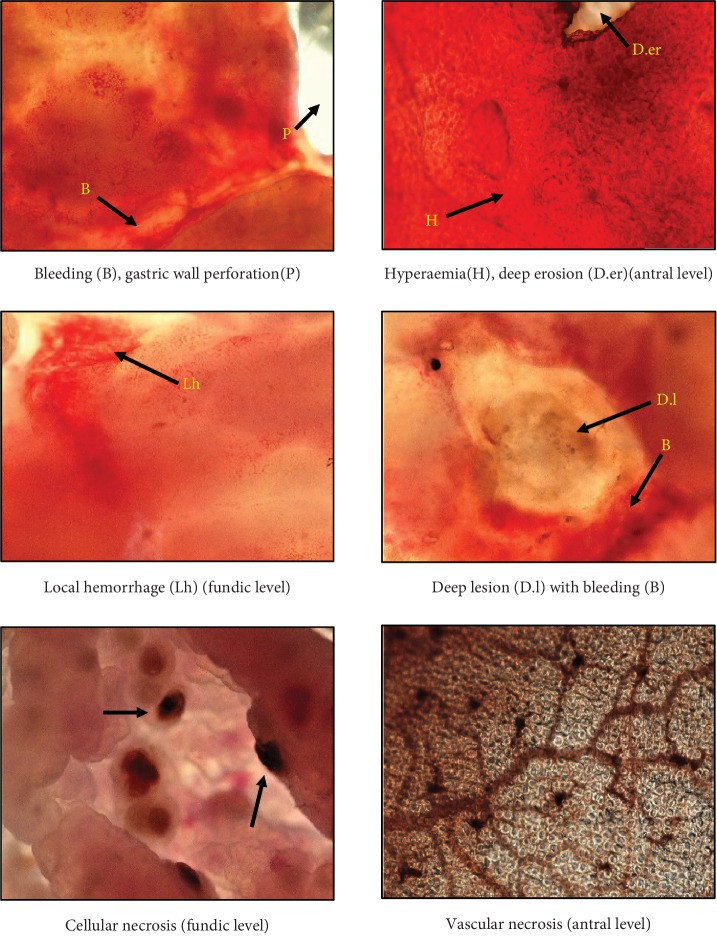
Microscopic assessment of the upper surface of ethanol-ulcerated gastric mucosae symptoms (10 × 40). Images represent a microscopic assessment of the symptoms observed on the upper surface of ethanol-ulcerated gastric mucosae like bleeding and cellular and vascular necrosis.

*(2) Microscopic Examination of the Upper Surface of Gastric Mucosa*. The observation of the upper surface of the stomachs of the different experimental groups showed that absolute ethanol induced necrosis in some zones, active vascular congestion, and local hemorrhages. In some areas, we could note erosions and even more ulceration reaching the deepest layers of the stomach wall at the limit of stomach perforation. The intensity of these symptoms was reduced by the pretreatments. The most potent preventive pretreatment was OFI oil dose 2, followed by OFI oil dose 1, ranitidine, and sucralfate in comparison to negative and positive controls (Figure 6).

**Figure 7:**
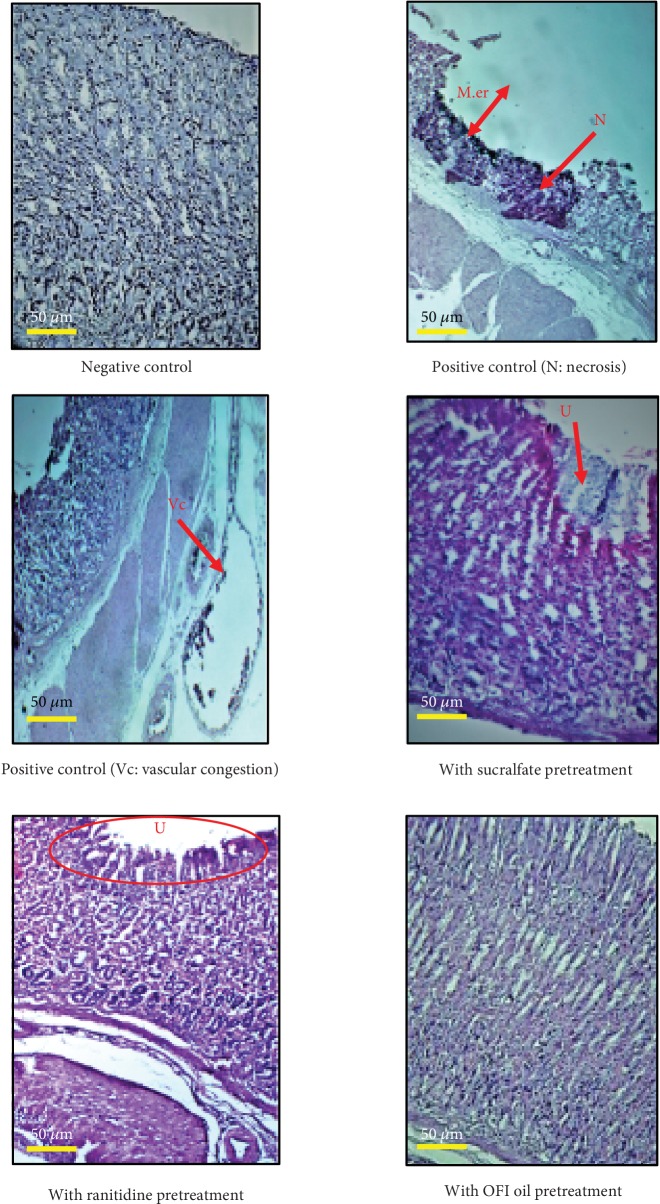
Microscopic assessment of H&E staining biopsies from gastric mucosa of the different experimental groups. M.er: mucosal erosion; N: necrosis; U: ulcer; Vc: vascular congestion. Images depict microscopic assessment of H&E staining biopsies collected from gastric mucosa from the experimental groups. Symptoms like mucosal ulcer, necrosis, and vascular congestion are indicated on the photographs.

*(3) Histological Investigation of Gastric Mucosal Biopsies*. Histological exploration of the H&E-stained biopsies collected from the stomachs of the different experimental groups revealed the following results:

1. Treatment with absolute alcohol in the positive group caused severe vascular congestion of the lamina propria at the esophageal-cardiac junction, cellular desquamation, and a discrete surface erosion of the mucosa with moderate infiltration of neutrophils. At the fundic level, the vascular congestion was more significant as it reached the submucosal and subserosal vessels. In addition, treatment with absolute alcohol caused significant acidophilic necrosis of the crypt layer. A desquamative degeneration of the cells of the deep glands has been observed. At the antral level, a vascular congestion caused by alcohol was as severe as that observed at the fundus level. Necrosis with extensive disbonding of the crypto layer has been caused, reaching even the muscular layer in some areas, associated with the degeneration of glandular cells
2. Pretreatment with sucralfate prior to the induction of gastric ulcer by absolute ethanol reduced the symptoms observed at the three levels mentioned above. At the esophageal-cardiac junction, sucralfate provided moderate mucosal protection. Superficial desquamation and slight vascular congestion were noted. At the fundic level, we observed superficial erosion of the surface epithelium, massive desquamation of cells at the glands and crypts, and discrete inflammatory infiltration by neutrophil polymorphs of the submucosa. At the antral level, we recorded clear desquamation of the glands as well as significant vascular congestions caused by absolute ethanol and infiltration with polymorphonuclear neutrophils and macrophages
3. At the esophageal-cardial junction level, the force-feeding of the rats with absolute ethanol after pretreatment of the gastric mucosa with ranitidine caused less damage than that in the negative control and sucralfate group. Minimal cardiac desquamation, low neutrophil infiltration, and slight vascular congestion have been nevertheless observed. At the fundic level, massive cellular desquamation, some superficial exulcerations, reduced vascular congestion, and moderate polynuclear infiltration were noted. At the antral level, we noticed some neutrophil infiltrates. However, the glands were preserved.
4. Histopathological examination of the H&E-stained biopsies collected from gastric mucosae from rats pretreated with OFI oil indicated that symptoms of ethanol-induced damages were minimal. Dose 2 oil (7 mL/kg/bw) was more efficient than dose 1 oil (3.5 mL/kg/bw). At the esophageal-cardial junction, epithelial cellular desquamations and neutrophil leukocyte infiltrations in the chorion were much reduced. At the fundic and antral levels, submucosal vessel congestion was reduced. A very low epithelial cellular desquamation accompanied by some superficial erosions have been observed. Some of these observations were presented in Figure 7

### 3.4. Healing Effect of OFI Oil of Ethanol-Induced Gastric Ulcer

**Figure 8:**
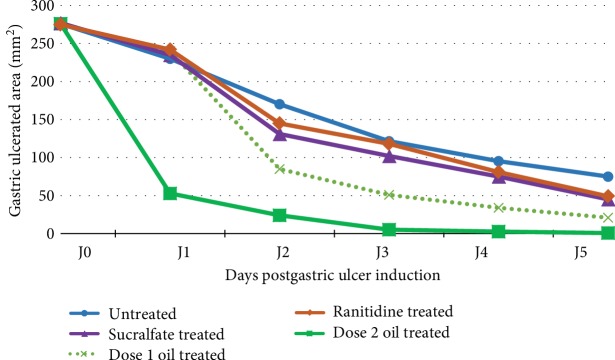
Healing rate evolution of the ulcerated areas during the five days postulcer induction in the different experimental groups. Image represents the evolution of the healing rate of the ulcerated surface of gastric mucosa over five days postulcer induction. Comparison between the different groups indicates that OFI oil dose 2 is the most efficient treatment to speed-up the healing process compared to OFI oil dose 1, ranitidine, and sucralfate.

Figure 8 depicts the evolution of the ulcerated area surface in mm^2^ over 5 days after inducing gastric ulcer. Curve analysis indicates the reduction of the surface of ulcerated areas in gastric mucosa during the treatment period. The healing process speeds were different. We noticed a similar evolution in untreated controls, sucralfate, ranitidine, and OFI oil dose1 during the first 24 hours postulceration. On the other hand, oral treatment with OFI oil dose 2 significantly reduced the ulcerated area by almost 80%. A very slight improvement was recorded with ranitidine compared to sucralfate and control from day 2. Dose 2 of OFI oil accelerated the peptic ulcer healing at a rate of 91% on day 2 compared to OFI oil dose 1 (72%), ranitidine (55%), sucralfate (50%), and untreated control (40%). At the 3rd day postalcohol intake, a healing rate of 99% was registered with OFI oil dose 2 and a healing rate of 80% was registered with OFI oil dose 1, while the gastric healing rates with ranitidine and sucralfate were quite similar to that of the untreated group (56%). Complete healing of ulcers was registered at the 4th day with OFI oil dose 2.

## 4. Discussion

Gastric ulcer is a disease that is spreading all over the world causing enhanced morbidity and mortality rates in humans. Its etiology is due to an imbalance between the protective and aggressive factors of the gastric mucosa. Allopathic medicines used in the treatment of this pathology have many side effects which can be very harmful. Therapeutics used in the treatment of gastric ulcer are based mainly on the use of proton pump inhibitors (PPIs) as well as drugs that reduce HCl production by acting on histamine H_2_-receptors and those that stimulate mucus synthesis or act as gastric dressings [66–68]. Alternative medicine research focuses on the finding of new therapeutic tools that would be efficient to prevent the occurring of gastric injuries or ulcerations and to heal them if they already occurred with minimal or no side effects.

The current study highlighted the antiulcerative protective and healing efficiency of OFI oil extracted by cold pressing against acute peptic ulcer induced by absolute ethanol, in comparison to two allopathic reference drugs, sucralfate and ranitidine. Our data have shown that OFI oil has significantly reduced the severe mucosal damages (lesions, ulcers, bleeding, and necrosis) observed in several areas in the gastric wall of starved rats then orally treated with absolute ethanol (positive control). We noted a major protection with dose 2 (7 mL/kg/bw) compared to dose 1 (3.5 mL/kg/bw) as it may give sufficient amounts of active biocompounds to be efficient. Gastric parameters were better normalized (*p* < 0.01). The surface of ulcerated areas was much reduced by dose 2 oil pretreatment (*p* < 0.001) vs. positive control and rats pretreated with reference drugs, thus strongly enhancing the percentage of ulcer inhibition. Mucus secretion has been stimulated, gastric juice pH was elevated, and its volume was reduced.

Pretreatment with the reference drugs had reduced the surface of the lesions and the necrosis areas in the gastric mucosa but the ulcerogenic effect of ethanol remained relatively strong. Ranitidine is a drug commonly administered to cure peptic ulcer and the gastroesophageal reflux disease (GERD) [66]. It acts as an antagonist of the H_2_-receptors reducing by this way the gastric mucosa acid secretion. Sucralfate is known as a gastroprotective agent that forms a layer on the gastric mucosa to act as a barrier against acidic secretions and irritating factors [67]. Our study had shown that the pretreatment with ranitidine slightly reduced the symptoms of peptic ulcer to a degree equivalent with that of sucralfate.

Our results are consistent with previous studies that have reported the ulcerative effect of orally applied ethanol on gastric mucosa [41–44, 46–48]. It induces disruptions in gastric mucosal integrity, epithelial cellular exfoliation, mucosal friability, inflammation, and local hemorrhage due to intensive microvascular changes. The noxious effects of ethanol were first attributed to the occurrence of an abnormal intense oxidative stress state in the gastric mucosa with increased reactive oxygen species (ROS) production. It has been proved that alcohol intake provoked an alleviation of the mitochondrial membrane potential, thus leading to a perturbation in the mitochondrial electron chain transfer and an overproduction of oxygen free radical O_2_^−^ [69, 70]. Moreover, ROS production is associated to the formation of lipid peroxidation products such as MDA and the depletion of cellular enzymatic and nonenzymatic antioxidative defense systems such as SOD, CAT, and GPx [47]. It has been reported that ethanol-induced gastric injuries were correlated to an enhancement of MDA secretion levels in the gastric mucosa [46]. Our data have shown that OFI oil exhibited high scavenging activity against free radicals DPPH and ABTS, as % of radical inhibition, respectively, 88.41 and 87.42 Vit. C eq/g oil, allowing it to counteract in a preventive way ethanol oxidative effects on gastric mucosal cells. The antioxidative potential of OFI oil could be attributed to its richness in active biocompounds such as polyphenols (46.71 Gallic acid eq/g oil), flavonoids (3.1 mg Quercetin eq/100 g oil), carotenoids (10.52 mg/kg oil), chlorophylls (4.57 mg/kg oil), and Vitamin E tocopherols (863.21 mg/kg oil) with an average of 797.8 mg/kg oil for the fractions *β*- and *γ*-tocopherols. Indeed, several studies have attributed the antioxidant potentials of these compounds against the harmful effects of free radicals that induce pathophysiological states such as cardiovascular diseases, diabetes, and degenerative disorders like dementias and Parkinson's disease [71–76]. Pretreatment of gastric mucosa with OFI oil provided it with notable amounts of nonenzymatic antioxidants, thus stimulating the mechanisms of cellular defense against oxidative stress.

The linear and punctual lesions inflicted to gastric mucosa by absolute ethanol intake could be due to a direct damaging effect on mucosal cell membranes and to indirect effects by the stimulation of inflammatory pathways with an upregulation of gene expression of proinflammatory cytokines such as TNF-*α*, IL-1-*β*, IL-6, IL-16, TGF-*β*1, NF-*κ*B (nuclear factor-kappa B), and iNOS (inducible nitric oxide synthase that generates the ulcerative NO via ROS production in gastric mucosal cells) and a downregulation of TGF-61 (Tumor growth factor-61) which actually regulates some signaling pathways including angiogenesis, cell proliferation, and extracellular matrix component differentiation [48]. TNF-*α* participates in gastric ulcer via promoting oxygen free radicals and enhancing apoptosis through the caspase cascade pathway and neutrophil migration into gastric mucosa facilitated by vessel congestion. Moreover, TNF-*α* is implicated in the activation of other intracellular inflammatory signaling pathways including INF-*γ* which upregulates its own production. Several studies have attributed the anti-inflammatory activities to plant sterols and especially to *β*-sitosterol as it displays inhibitory effects on proinflammatory agents such as IL-6 and TNF-*α* by monocytes [76, 77]. Furthermore, our phytochemical analysis of OFI oil has shown that it is rich in sterols especially in *β*-sitosterol (81.280 g/100 g of total sterols) and campesterol (11.043 g/100 g of total sterols). These compounds could further explain the antioxidant and the anti-inflammatory properties of this oil as a pretreatment in the protection of the gastric mucosa against ulcer. In fact, we could note a significant reduction of inflammatory leukocyte infiltration into mucosa and submucosa layers of the gastric wall.

Furthermore, ethanol evoked an overproduction of HCl by parietal cells, thus reducing gastric juice pH and enhancing gastric juice volume. However, OFI oil pretreatment especially with dose 2 significantly reduced gastric juice volume and acidity, thus minimizing acid aggression of the mucosa. HCl secretion takes place in the parietal cells of the oxyntic glands following stimulation by gastrin, produced by the endocrine G cells of the glands. Histamine secreted by ECL (enterochromaffin-like) cells from the fundic glands contributes to the stimulation of mucosal parietal cells [12].

The significant increase in pH of gastric juice and mucus weight (*p* < 0.001) that we recorded after ulcer induction by absolute ethanol in rats pretreated with prickly pear oil (especially with dose 2) vs. negative control and sucralfate and ranitidine groups could suggest an inhibitory activity of this oil on H_2_-histaminic or on the other stimulatory pathways including gastrin and/or acetylcholine parietal cell receptors. In addition, OFI oil components could act on cellular pathways in Goblet cells whose main role is mucus secretion but also bicarbonate (HCO_3_^−^) which has an alkaline pH, in order to protect the gastric mucosa from the aggressive effects of HCl and pepsin. Furthermore, it has been reported that [78] prostaglandins (PGEs) and in particular prostaglandin E2 (PGE2) exhibit a gastroprotective effect by positively influencing mucosal integrity defense systems. They are able to enhance epithelial cell resistance to injury induced by cytotoxins and to stimulate mucus and bicarbonate synthesis and secretion. PGE synthesis depends mainly on the activity of two isoenzyme cyclooxygenases (COX), i.e., COX-1 and COX-2, that are involved in the synthesis pathway of arachidonic acid-derived eicosanoids (leukotrienes, thromboxanes, and prostaglandin). It has been shown that *β*- and *γ*-tocopherols are able to inhibit their activities and that of 5-lipoxygenase (5-LOX) that catalyzes leukotriene (LT) biosynthesis, thus limiting inflammatory symptoms induced by absolute ethanol on gastric mucosa [79]. Since OFI oil is very rich in *β*- and *γ*-tocopherol fraction Vitamin E, they may inhibit COX-1 and COX-2, leading indirectly to the enhancement of PGE2 and so to an increase in mucus production and mucosa protection. Moreover, the fatty acid analysis of OFI oil has shown that the major components are linoleic acid (C18 : 2 *n*-6), oleic acid (C18 : 1 *n*-9), and palmitic acid (C16 : 0) with, respectively, the rates of 61.63, 21.18, and 12.24 g/100 g of total free fatty acids. As described by previous studies, at sufficient levels, and in addition to their role in the construction and consolidation of cell membranes, these fatty acids are involved in anti-inflammatory cellular processes by inhibiting proinflammatory cytokines (TNF-alpha and IL-6) as well as COX-1 and COX-2 [80]. This is able to enhance mucus secretion and reduce HCl production by the cells of the gastric mucosa under the effect of a sufficient dose of OFI oil.

On one hand, our current study has demonstrated that OFI oil exhibited a strong healing effect of ethanol-induced gastric ulcer. The comparison between all experimental groups showed that gastric ulcer could heal naturally without treatment but in a slow mode. The healing process has been accelerated in ascending mode by ranitidine then sucralfate, OFI oil dose 1, and finally by OFI oil dose 2. The evolution of the ulcerated area during five days after inducing ulcer indicated a speeding-up of the healing process compared to the controls and to the reference drug-treated rats. In a previous work [53], we have demonstrated that OFI oil extracted under cold pressing has a high potential in the healing of full-thickness skin wounds in rats as well as in antimicrobial activities against bacteria, yeast, and fungi. We have noted an acceleration of the healing process compared to control and to a reference healing drug.

It should be remembered that the healing process is a very important phenomenon in restoring the integrity of the external (skin) and internal (mucous membranes) barriers of the human body. Any injury can be a gateway to germs and certain harmful or toxic substances.

The gastric ulcer healing process is an innate, genetically programmed injury repair of gastrointestinal mucosae. It includes overlapping phases: stopping blood loss if bleeding has occurred, inflammation, cell proliferation, reepithelialization and angiogenesis, granulation tissue formation, and extracellular matrix reconstitution and remodeling, as well as the reestablishment of gastric glands and other mucosal components [3]. As described above, our results revealed that intragastric force-feeding with ethanol caused severe necrotizing linear lesions with the loss of substance in gastric mucosa that affected even the submucosal layers, accompanied by vascular congestions, bleeding, and inflammatory infiltrations. Histologically, gastric ulcer has been described as two juxtaposed structures that complement each other to heal the wound: the base and the margin. It has been elucidated that the process of healing mucosal wounds is carried out through the action of a plethora of mediators and cytokines that are spilled and concentrated in the injured areas. The expression of their genes is induced in a well-synchronized spatial and time-delayed manner [81].

Cellular and molecular events occur in the ulcer margin (mainly in epithelial cells), while other events occur in its base (mesenchymal cells and extracellular matrix). For instance, EGF-R, c-fos, c-jun, egr-1, Sp-1, TFF-2/SP, PDGF, EGF, VEGF, HGF, bFGF, KGF, and TGF-*β* expression is triggered to activate, via several mechanisms such as autocrine and/or paracrine mechanisms, the epithelial cell migration of cells from the edges of the wound onto granulation tissue, to ensure reepithelialization. Moreover, cells lining gastric glands in the margin zone form tubes then undergo proliferation and differentiation to reconstruct damaged glands and mucosal crypts. At the base of the gastric ulcer, neoangiogenesis gets activated especially by VEGF, which stimulate endothelial cell migration, proliferation, differentiation, and capillary formation, thus restoring blood flow and providing nutrients and oxygen to the proliferating and differentiating epithelial and mesenchymal cells. Furthermore, it has been indicated that bone marrow-derived stem cells are able to migrate into the injured gastric mucosa and to differentiate in epithelial, endothelial, and glandular cells, contributing by this way to repopulating the human gastrointestinal tract [82].

OFI oil in sufficient amounts would on one hand have accelerated the ulcer healing rate by ensuring a protective lipid layer to the wound site against dehydration (like a dressing covering the mucosal crypts in a mimetic way such as sucralfate), thus promoting cytokine, growth, and transforming factor actions and on the other hand positively affected signaling pathways to restore the balance between aggressive and protective agents to regenerate mucosal components. This could be attributed to OFI oil antioxidants previously mentioned. The anti-inflammatory activity of phytosterols, especially *β*-sitosterol and PUFA, could corroborate the acceleration of ulcer healing by OFI oil. In an advanced stage, the reduction of acidity and gastric juice secretion under the effect of OFI oil would promote ulcer healing by filling then remodelling the extracellular matrix with structural proteins such as collagen types I, II, and III and elastin, proteoglycans, and hyaluronans. Reepithelialization, revascularization, and reconstruction of the glands and crypts covered by oil compounds, especially its unsaturated free fatty acids, would ensure the rapid restoration of the cytoarchitecture mucosa and the other layers of the gastric wall.

## 5. Conclusion

Our current findings suggest that *Opuntia ficus indica* seed oil extracted under cold pressing exhibited potent antioxidant, prophylactic, and therapeutic potentials against acute gastric ulcer induced by absolute ethanol. This is due to its richness in beneficial biocompounds which act in synergistic and complementary ways to ensure gastroprotection as well as gastric mucosal ulcer healing.

## Figures and Tables

**Table 1:** Physicochemical characteristics of OFI oil.

Parameters	
Physical state at room temperature	Liquid
Color	Greenish yellow
Odor	Slightly fruity
Property	Dry oil
Texture	Noncomedogenic
Density at 20°C (mass/volume)	0.905 ± 0.001
Density by gravimetry at 20°C	0.931 ± 0.010
Refractive index at 20°C	1.475 ± 0.001
Acid index	1.952 ± 0.034
Peroxide index (meq O_2_/kg of oil)	2.230 ± 0.061
Iodine index (g I_2_/100 g of oil)	108.52 ± 0.250
Saponification index (mg of KOH/g oil)	171.40 ± 0.430

**Table 2:** Free fatty acid composition of OFI oil.

	Content (g/100 g of total fatty acids)
Fatty acids	
C14 : 0	0.100 ± 0.005
C16 : 0	12.243 ± 0.023
C16 : 1 *n*-7	0.770 ± 0.015
C17 : 0	0.040 ± 0.005
C17 : 1 *n*-7	0.126 ± 0.003
C18 : 0	3.340 ± 0.030
C18 : 1 *n*-9	21.183 ± 0.064
C18 : 2 *n*-6	61.637 ± 0.068
C 18 : 3 *n*-3	0.233 ± 0.003
C20 : 0	0.233 ± 0.006
C22 : 0	0.093 ± 0.006
Fatty acid groups	
SFA	16.05 ± 0.03
UFA	83.95 ± 0.03
UFA/SFA	5.23 ± 0.11
PUFA	61.87 ± 0.06
MUFA	22.08 ± 0.04
PUFA/UFA (%)	73.69 ± 0.06
Cox value	6.61 ± 0.005

SFA: saturated fatty acids; UFA: unsaturated fatty acids; PUFA: polyunsaturated fatty acids; MUFA: monounsaturated fatty acids; UFA/SFA: unsaturated fatty acid to saturated fatty acid ratio; PUFA/UFA: polyunsaturated fatty acid to unsaturated fatty acid ratio; values given are the means of three measurements±standard error.

**Table 3:** Triacylglycerol (TAG) composition and positional distribution of fatty acids in OFI oil.

ECN (42)		TAG (g/100 g of total TAG)	
ECN 42	24.8633 ± 0.026	LLL	24.65 ± 0.029
		PoLL	0.213 ± 0.003

ECN 44	39.77 ± 0.012	OLL	22.246 ± 0.015
		PLL	17.523 ± 0.003

ECN 46	22.2267 ± 0.007	OOL	9.23 ± 0.006
		PoOO	3.056 ± 0.007
		SLL	9.94 ± 0.006

ECN 48	10.9767 ± 0.009	PLP	3.216 ± 0.003
		OOO	4.453 ± 0.003
		SOL	1.656 ± 0.003
		POO	1.646 ± 0.007

ECN 50	2.1633 ± 0.003	POP	1.416 ± 0.003
		SOO	0.126 ± 0.003
		AOO	0.62 ± 0.006

^∗^Values given are the means of three measurements ± standard deviation. A: arachidic acid; L: linoleic acid; O: oleic acid; P: palmitic acid; Po: palmitoleic acid; S: stearic acid.

**Table 4:** Phytosterol composition of OFI oil.

Sterol fractions	Content (% of total sterols)
*β*-Sitosterol	81.280 ± 0.115
Campesterol	11.043 ± 0.185
*Δ*-7-Avenasterol	2.210 ± 0.005
Stigmasterol	1.576 ± 0.185
Clerosterol	1.417 ± 0.003
*Δ*-7-Stigmastenol	1.353 ± 0.003
*Δ*-5.24-Stigmastadienol	0.963 ± 0.067
Cholesterol	0.157 ± 0.003
Brassicasterol	0.000 ± 0.000

^∗^Values given are the means of three measurements ± standard error.

**Table 5:** Vitamin E tocopherol composition in OFI oil.

Tocopherol fractions	Content (mg/kg oil)	% total tocopherols
*α*	11.49 ± 0.27	1.33
*β* + *γ*	797.8 ± 0.79	92.42
*δ*	53.92 ± 0.46	6.24
Total tocopherols	863.21 ± 1.16	—

^∗^Values given are the means of three measurements ± standard error.

**Table 6:** Total phenolics, flavonoids, carotenoids, and total chlorophyll contents.

Compounds	Content
Total phenolics (Gallic acid eq/g oil)	26.50 ± 0.00
Flavonoid mg Quercetin eq/g oil	3.1 ± 0.25
Carotenoid (mg/kg)	10.52 ± 0.005
Total chlorophylls (mg/kg)	4.57 ± 0.001

^∗^Values are presented as means of three measurements ± standard error.

**Table 7:** Scavenging activity against free radical DPPH and ABTS of OFI oil.

Free radical	% inhibition (Vit. C eq/g oil)
DPPH	88.410 ± 0.59
ABTS	87.420 ± 0.11

## Data Availability

The data used to support the findings of this study are available from the corresponding author upon request.
